# The Antioxidant Activity of a Prepared Cream Containing Saffron Extract and Grape Seed Extract

**DOI:** 10.1155/bmri/4463017

**Published:** 2026-04-24

**Authors:** Wissam Zam, Aziz Alkhaddour, Samer Housheh

**Affiliations:** ^1^ Department of Analytical and Food Chemistry, Faculty of Pharmacy, Tartous University, Tartous, Syria, tartous-univ.edu.sy; ^2^ Lab of Bio Regeneration Technology, Southern Federal University, Rostov, Russia, sfedu.ru; ^3^ Department of Quality Control and Pharmaceutical Chemistry, Faculty of Pharmacy, Wadi International University, Homs, Syria, wiu.edu.sy

**Keywords:** antioxidant cream, grape seed extract, oxidative stress, physicochemical evaluation, saffron extract

## Abstract

This study evaluates the antioxidant activity of a topical water‐in‐oil cream formulated with saffron (*Crocus sativus*) and grape seed (*Vitis vinifera*) extracts and characterizes its physicochemical properties. HPLC profiling confirmed that grape seed extract was rich in catechins (26.7%), epicatechins (21.9%), and procyanidins B1 and B2 (13.6% and 9.5%), while saffron extract contained high levels of trans‐4‐GG‐crocin (28.4%), trans‐2‐gg‐crocin (23.5%), and picrocrocin (13.4%). In vitro assays using human lymphocyte cultures demonstrated that saffron extract (2.5%) reduced fast‐flash chemiluminescence from 36.1 × 104 to 14.3 × 104, while grape seed extract (5.0%) decreased the same parameter to 16.3 × 104. Light‐sum ROS output similarly declined by 39%–50% across extract concentrations. Gene expression analysis revealed significant upregulation of SOD1, NFE2L2, and JUN, indicating activation of endogenous antioxidant pathways. The final cream formulation, containing 2.5% saffron and 5.0% grape seed extract, exhibited a stable pH of 5.9, viscosity of 31,869 cP, and spreadability of 24.32 g/cm^3^/s, with no observed irritancy during application tests. These results demonstrate that combining crocin‐rich saffron and proanthocyanidin‐rich grape seed extracts have a synergistic antioxidant effect and can be successfully incorporated into a stable, skin‐compatible topical preparation. The formulation shows promise as a dermal antioxidant therapy aimed at reducing oxidative stress and supporting skin rejuvenation.

## 1. Introduction

The imbalance between reactive oxygen species (ROS) generation and antioxidant defenses is increasingly recognized as a driving factor in many diseases, ranging from premature skin aging to chronic inflammation and neurodegenerative disorders [1–4]. ROS—including superoxide, hydroxyl radicals, and hydrogen peroxide—arise naturally during mitochondrial activity and enzymatic reactions. At controlled levels, they function as signaling molecules that influence cell growth, immunity, and gene regulation [5]. When their production surpasses the neutralizing capacity of antioxidant systems, oxidative injury to DNA, proteins, and lipids occurs, ultimately impairing cellular and tissue function [6–8].

To counteract oxidative stress, cells rely on a complex defense system composed of both enzymes and small molecules. Key enzymatic antioxidants include superoxide dismutase (SOD), catalase (CAT), and glutathione peroxidase (GPx), while non‐enzymatic agents such as vitamins C and E, glutathione, and uric acid provide additional protection [9–11]. Together, these elements preserve redox balance by neutralizing reactive species. Central to this regulation is the Nrf2–ARE signaling pathway, which activates cytoprotective genes once Nrf2 enters the nucleus and binds to antioxidant response elements. Genes such as SOD1, HO‐1, and glutathione S‐transferases are among its primary targets [12, 13]. Moreover, Nrf2 interacts with other transcription factors, including AP‐1 and NF‐*κ*B, thereby linking antioxidant responses with inflammatory signaling [14].

Because the body’s natural antioxidant defenses are often insufficient, researchers have turned to plant‐derived compounds as complementary strategies against oxidative stress. Diverse phytochemicals—including polyphenols, flavonoids, carotenoids, and terpenoids—are known for their ability to quench free radicals and influence redox‐sensitive signaling pathways [15–17]. Two extracts of particular interest are saffron (*Crocus sativus*) and grape seed (*Vitis vinifera*). Saffron stigmas contain bioactive molecules such as crocin, crocetin, picrocrocin, and safranal, which have been linked to antioxidant, anti‐inflammatory, and neuroprotective activities in both cellular and animal studies [18–20]. Crocin, particularly trans‐4‐GG‐crocin and trans‐2‐GG‐crocin, are potent radical scavengers capable of reducing oxidative damage to lipids, proteins, and DNA. These compounds have been shown to activate the Nrf2–ARE pathway, leading to increased transcription of antioxidant genes such as SOD1 and HO‐1 [21]. Crocetin also modulates inflammatory signaling by suppressing NF‐*κ*B activation [22], while picrocrocin and safranal contribute additional antioxidant and cytoprotective effects [23]. Together, these constituents make saffron a strong candidate for topical applications aimed at mitigating oxidative stress in skin tissue. Grape seed extract provides a rich source of proanthocyanidins, polyphenols with stronger radical‐scavenging activity than vitamins C and E [24, 25]. Beyond direct ROS neutralization, these compounds also stimulate endogenous defense pathways, notably through Nrf2‐dependent gene regulation [26]. Proanthocyanidins are known to activate Nrf2‐dependent transcription and enhance endogenous antioxidant defenses [27], while also modulating MAPK/ERK signaling pathways that regulate AP‐1 components such as JUN [28]. Their combined antioxidant and anti‐inflammatory properties have been demonstrated in leukocyte cultures, animal models, and dermatological studies, supporting their use in formulations targeting oxidative skin damage [29].

In recent years, dermatology and cosmetic science have increasingly focused on the topical application of antioxidants. As the body’s outermost defense, the skin is continuously exposed to environmental stressors such as UV radiation and pollutants, making it particularly vulnerable to oxidative damage. Elevated ROS levels in cutaneous tissue accelerate collagen breakdown, weaken barrier integrity, disrupt pigmentation, and drive premature aging processes [30–32]. Incorporating plant‐derived antioxidants into creams and lotions provides a potential means of restoring redox balance and supporting tissue repair. The effectiveness of such formulations, however, depends not only on the bioactivity of the extracts but also on pharmaceutical attributes such as stability, pH, viscosity, spreadability, and compatibility with the skin [33].

Although several researches’ outcomes underscore the therapeutic promise of natural antioxidants, their application in dermatological formulations has not been fully realized. Specifically, there is a lack of studies that integrate saffron and grape seed extracts into one topical preparation and assess both its molecular antioxidant activity and its pharmaceutical performance. In light of these gaps, our study pursued two primary aims. The first was to develop a stable water‐in‐oil cream incorporating saffron and grape seed extracts and to characterize its physicochemical properties relevant to topical use. The second was to assess the antioxidant activity of this formulation in vitro, focusing on its capacity to lower ROS levels and influence the transcription of key redox‐responsive genes (SOD1, NFE2L2, and JUN) in human lymphocyte cultures. By combining phytochemical analysis, molecular assays, and formulation science, this work provides a basis for designing antioxidant creams that may enhance skin protection and support rejuvenation.

## 2. Materials and Methods

### 2.1. Extracts Preparation

Concentrations of the plant extracts were selected following preliminary trials that assessed their impact on cell viability and mitotic activity across a range of 0.5%–10%. Based on these observations, optimal levels were identified and applied in subsequent experiments.

### 2.2. Preparation of Grape Seed Extract

Grape seed was purchased from a local market in Tartous, Syria. The extract was obtained by incubating 20 g of seed powder with 200 mL of 70% ethanol at 50°C and 120 rpm for 24 h. The mixture was filtered through Whatman No. One paper, and the extraction cycle was repeated up to three times to maximize recovery of polyphenolic compounds [25]. The combined filtrates were concentrated using a rotary evaporator at 45°C for 2 h. Extracts were stored at refrigerator in airtight, amber‐colored containers with polyseal caps to prevent degradation.

### 2.3. Preparation of Saffron Extract

Saffron was cultivated locally at Wadi International University for research purposes. The extract was prepared by combining 5 g of saffron with 100 mL of 50% ethanol and incubating the mixture at 25°C for 5 h. The solution was passed through Whatman No. One paper to separate the dissolved constituents. To maximize recovery of polyphenolic compounds, the extraction was repeated up to three times [26]. The pooled filtrates were concentrated using a rotary evaporator at 45°C for 2 h. Extracts were stored at refrigerator in airtight, amber‐colored containers with polyseal caps to prevent degradation.

### 2.4. Phytochemical Screening

#### 2.4.1. Analysis of Grape Seed Extract by High Performance Liquid Chromatography (HPLC)

The analysis was carried out according to Krasteva et al. using C18 column (4.6 × 250 mm, particles size of 5 *μ*m) and a photodiode array (PDA) detector at a flow rate of 1 mL/min. The mobile phase consists of two eluents: eluent A = 0.1% phosphoric acid in deionized water and eluent B = acetonitrile. The procedure is made up of an eluent gradient according to the following program: 0–5 min 90% A‐10% B; 5.1–20 min 80% A‐20% B; 20.1–25 min 75% A‐25% B; and 25.1‐50: 70% A‐30% B. The PDA wavelengths range was from 210 to 500 nm, the column temperature was kept at 24°C, and the injection volume was 10 *μ*L [34].

#### 2.4.2. Analysis of Saffron Extract by HPLC

The analysis was carried out according to Ruggieri et al. using C18 column (4.6 × 250 mm, particles size of 5 *μ*m) and a PDA detector at a flow rate of 1 ml/min. The mobile phase consists of two eluents: eluent A = deionized water and eluent B = acetonitrile. Initial conditions were set at 95% A and 5% B, reaching 95% B within 30 min, and returning to initial conditions within 20 min for a total run time of 50 min. The PDA wavelengths range was from 250 to 450 nm, the column temperature was kept at 24°C, and the injection volume was 10 *μ*L [35].

### 2.5. Lymphocyte Culture

One hundred and fifteen Syrian participants (61 men and 54 women), aged between 22 and 29 years contributed to the study. All of them were healthy and did not suffer from chronic or acute diseases. All practical safety requirements in accordance with ethics were discussed and agreed upon by all research participants and obtained in consent forms following approval from the Bioethics Committee at Wadi International University (Approval No. 128/N.B.A dated 19/03/2025). Peripheral blood lymphocytes were isolated and cultured under standard conditions as described previously [30]. Cells were maintained in RPMI 1640 medium supplemented with fetal bovine serum and mitogen. Saffron extract added at 1.25% or 2.5% (w/v) and grape seed extract at 3.75% or 5.0% (w/v). After 72 h of incubation, cell pellets obtained by centrifugation were used for subsequent gene expression analysis. All experiments were performed using three independent biological replicates, and each measurement was conducted in technical triplicate.

### 2.6. Luminol‐Amplified Chemiluminescence (CL) Method for Assessing Oxidative Stress

ROS levels were assessed using the luminol‐amplified chemiluminescence assay, as previously described [30, 31]. The method detects ROS‐dependent light emission, with parameters such as fast flash intensity and total light output recorded using a photochemistry analyzer. In this study, test samples containing saffron extract (1.25% or 2.5% w/v) and grape seed extract (3.75% or 5.0% w/v) were analyzed, and CL indices were calculated relative to background illumination. All experiments were performed using three independent biological replicates, and each measurement was conducted in technical triplicate.

### 2.7. Gene Expression Measurement

Total RNA was extracted from lymphocyte pellets and reverse‐transcribed into cDNA following standard protocols [32]. Quantitative real‐time PCR was performed using primers targeting GAPDH, Nrf2, JUN, and SOD1 (Table 1) as previously described in the literature [32]. Relative gene expression was calculated using the *ΔΔ*Ct method [33], with GAPDH serving as the internal control. The effects of saffron extract (1.25% and 2.5% w/v) and grape seed extract (3.75% and 5.0% w/v) on transcriptional activity were compared against untreated controls. All experiments were performed using three independent biological replicates, and each measurement was conducted in technical triplicate.

**Table 1:** Sequences of primers and probes for PCR.

Gene	Sequence of primers and probes
SOD1	F: 5 ^′^‐ACTGGTGGTCCATGAAAAAGC‐3 ^′^
	R: 5 ^′^‐AACGACTTCCAGCGTTTCCT‐3 ^′^
	Probe Fam‐CCGATGTGTCTATTGAAGATTCTG‐BHQ
Nrf2	F: 5 ^′^‐CAGCGACGGAAAGAGTATGA‐3 ^′^
	R: 5 ^′^‐TGGGCAACCTGGGAGTAG‐3 ^′^
	Probe 5 ^′^‐FAM CTCATGTCCATCATGGAAATGCAGGCTAMRA‐3 ^′^
JUN	F: 5 ^′^‐GTTGCGGCCGCGAAACTT‐3 ^′^
	R: 5 ^′^‐CATTGCCCTCGAGCCCTG‐3 ^′^
	Probe Fаm‐CTCGCCCACGCAGGAGCTTC
GАPDH	F 5 ^′^‐АGGTCGGАGTCААCGGАTTT‐3 ^′^
	R 5 ^′^‐АTCGCCCCАCTTGАTTTTGG‐3 ^′^
	Probe Fаm‐GGCGCCTGGTCАCCАGGGCT‐BHQ1

### 2.8. Statistical Analysis

Data represent the mean ± standard deviation of three biological replicates, with each assay performed in technical triplicate. Gene expression data were normalized to GAPDH and analyzed relative to untreated controls. Relative transcript abundance was calculated using the *Δ*Ct method [36], and fold changes were determined by the 2^^–*ΔΔ*Ct^ approach of Livak and Schmittgen [33]. To confirm statistically significant differences between the sample ranges, the Mann–Whitney *U* test was used.

### 2.9. Preparing a Cream Containing Saffron Extract and Grape Seed Extract

A water/oil cream containing saffron extract and grape seed extract was prepared according to the ingredients listed in Table 2.

**Table 2:** Ingredients and quantities in grams required to prepare 100 g of a water/oil cream containing saffron extract and grape seed extract.

	Ingredients	Amounts in grams
Cold phase	Saffron extract	2.5
	Grape seed extract	5
Oil phase	Beeswax	15
	Stearic acid	10
	Cetostearyl alcohol	7.5
	Cetyl alcohol	7.5
	Paraffin oil	20
	Span 80	2
	Propylparaben	0.001
Aqueous phase	Tween 80	2
	Triethanolamine	4
	Agro gel	1
	Glycerin	5
	Methylparaben	0.01
	Water	Q.S 100 g

The preparation of the water‐in‐oil cream involves heating the oil phase in a water bath to 70°C, while separately heating the aqueous phase to 65°C. Once both phases reach their respective temperatures, the aqueous phase is gradually added to the hot oil phase with continuous stirring in one direction to ensure proper emulsification. The mixture is then removed from the water bath and stirred until it cools to 35°C. Finally, the cream is packaged in opaque, light‐resistant containers at a temperature not exceeding 25°C to preserve its stability and protect it from light‐induced degradation.

### 2.10. Evaluation of the Physicochemical Properties of the Cream

The cream was assessed for sensory attributes (color, odor, consistency, and physical state), irritancy, washability, phase stability, pH, viscosity, and spreadability using established protocols [37–39]. Irritancy was evaluated by topical application on human skin, while washability was tested with tap water. Phase separation was monitored during storage for 3 months under controlled conditions. pH was measured after dispersion in distilled water, viscosity determined with a rotary viscometer, and spreadability assessed using the slip‐weight method. These evaluations confirmed the formulation’s stability, skin compatibility, and ease of application.

## 3. Results and Discussion

The individual components in the grape seed extract were determined by HPLC/PDA. Under the described HPLC conditions, the eluted components were presented in Table 3. The extract was found to be highly rich in phenolic acid (gallic acid) and phenolic compounds, as monomeric flavan‐3‐ols (catechin, epicatechin) and their oligomeric proanthocyanidins, specifically procyanidins B1 and B2, reflecting the high tannin content typically associated with grape seeds.

**Table 3:** HPLC determination of phenolic compounds in grape seed extract.

Compound	% area/total area
Gallic acid	12.3
Catechins	26.7
Ellagic acid	1.3
Epicatechins	21.9
Procyanidins B1	13.6
Procyanidins B2	9.5
Procyanidins B3	1.5

Saffron extract exhibited a characteristic chromatographic profile dominated by crocin esters, accompanied by picrocrocin, safranal, and crocetin. The major trans‐crocins (4‐GG, 2‐gg, and 3‐Gg) accounted for the largest proportion of the total peak area, reflecting the typical pigment composition responsible for saffron’s intense coloration. A smaller contribution from the cis‐isomer (cis‐2‐G‐crocin) was also observed, indicating limited photo‐ or thermal isomerization. The detailed percentage distribution of these constituents was presented in Table 4.

**Table 4:** HPLC determination of phenolic compounds in saffron extract.

Compound	% area/total area
Trans‐4‐GG‐Crocin	28.4
Trans‐2‐gg‐Crocin	23.5
Trans‐3‐Gg‐Crocin	11.7
Cis‐2‐G‐Crocin	2.7
Picrocrocin	13.4
Safranal	2.8
Crocetin	2.4

The present study demonstrated that saffron and grape seed extracts possess significant antioxidant activity when applied to human lymphocyte cultures, and that their incorporation into a topical cream yields a formulation with favorable physicochemical properties. Both extracts reduced luminol‐dependent chemiluminescence (Table 5, Table 6), indicating a decrease in ROS generation, and modulated the transcription of redox‐sensitive genes, including SOD1, JUN, and NFE2L2. These findings support the potential of combining phytochemicals with complementary antioxidant mechanisms into a single dermal preparation.

**Table 5:** Effect of saffron extract on luminol‐amplified CL levels in peripheral blood leukocyte culture.

CL index	Control	Extract concentrations	CL index value	*p* value∗
Fast flash (counts per second)	36.1∗10^4^ ± 7.0∗10^4^	1.25%	20∗10^4^ ± 3.3∗10^4^	0.053
		2.5%	14.3∗10^4^ ± 2.9∗10^4^	0.01
Light sum (millions of counts per 100 seconds)	18.6∗10^6^ ± 2.6∗10^6^	1.25%	11.3∗10^6^ ± 2.5∗10^6^	0.059
		2.5%	9.2∗10^6^ ± 2.3∗10^6^	0.04

Note: *p* value∗—compared with control.

**Table 6:** Effect of grape seed extract on luminol‐amplified CL levels in peripheral blood leukocyte culture.

CL index	Control	Extract concentrations	CL index value	*p* value∗
Fast flash	36.1∗10^4^ ± 7.0∗10^4^	3.75%	20∗10^4^ ± 3.3∗10^4^	0.053
		5.0%	16.3∗10^4^ ± 2.9∗10^4^	0.01
Light sum	18.6∗10^6^ ± 2.6∗10^6^	3.75%	11.3∗10^6^ ± 2.5∗10^6^	0.059
		5.0%	11.2∗10^6^ ± 2.3∗10^6^	0.04

Note: *p* value∗—compared with control.

Our results are in agreement with previous studies that have investigated the biological activity of grape seed extract. In particular, Mashkina and Alkhaddour [40] reported that grape seed and other phytochemical extracts modulated cytokine gene expression in human leukocytes, highlighting their immunomodulatory and antioxidant potential. The present work extends those findings by demonstrating that grape seed extract, when combined with saffron, not only reduces ROS levels but also influences transcription of redox‐sensitive genes in lymphocyte cultures. Moreover, unlike the earlier study, we translated these molecular observations into a topical cream formulation and evaluated its pharmaceutical properties, thereby bridging cellular assays with practical dermal application.

**Figure 1:**
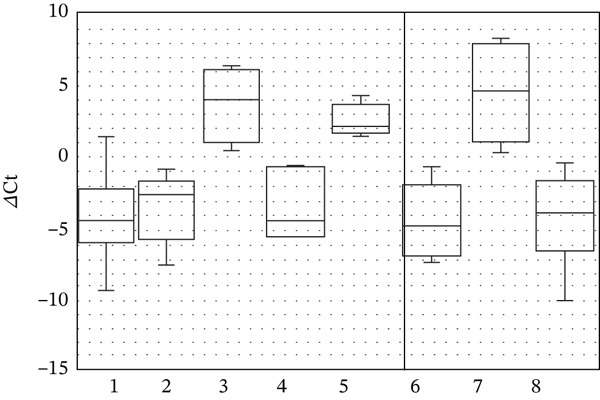
SOD1 gene transcription level in human blood cell culture medium relative to GAPDH gene transcription in the presence of plant extracts in the culture medium (1—control; 2—grape seed extract 3.75%; 3 and 4—grape seed extract 5.0%; 5 and 6—saffron extract 1.25%; 7 and 8—saffron extract 2.5%). Values represent mean ± SD of three biological replicates, each measured in technical triplicate. Statistical significance was determined using the Mann–Whitney *U* test (*p* < 0.05).

**Figure 2:**
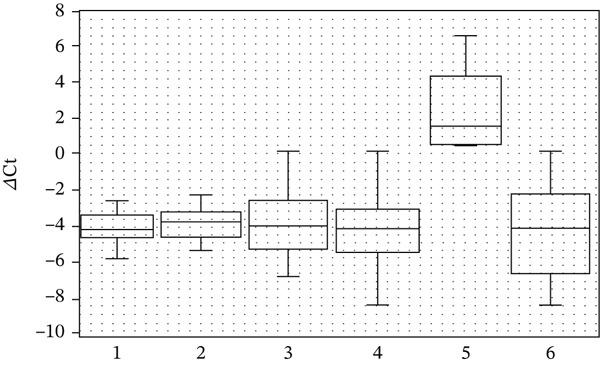
The transcription level of NFE2L2 gene in human blood cell culture medium relative to GAPDH gene transcription in the presence of plant extracts in the culture medium (1—control; 2—grape seed extract 3.75%; 3—grape seed extract 5.0%; 4—saffron extract 1.25%; 5 and 6—saffron extract 2.5%). Values represent mean ± SD of three biological replicates, each measured in technical triplicate. Statistical significance was determined using the Mann–Whitney *U* test (*p* < 0.05).

The antioxidant effect of saffron extract observed here is consistent with reports attributing its activity to crocins and other carotenoids [41–43]. Crocin 1 (*α*‐crocin), the most abundant carotenoid in saffron, has been shown to scavenge free radicals and protect cellular components from oxidative damage. In our study, saffron extract significantly increased SOD1 transcription (Figure 1) and enhanced NFE2L2 expression (Figure 2), confirming its role in activating endogenous antioxidant defenses. Similarly, grape seed extract, rich in proanthocyanidins, demonstrated strong antioxidant capacity, reducing ROS levels by more than twofold at the higher concentration tested. This is in agreement with earlier studies showing that proanthocyanidins are more effective radical scavengers than vitamins C and E [44].

**Figure 3:**
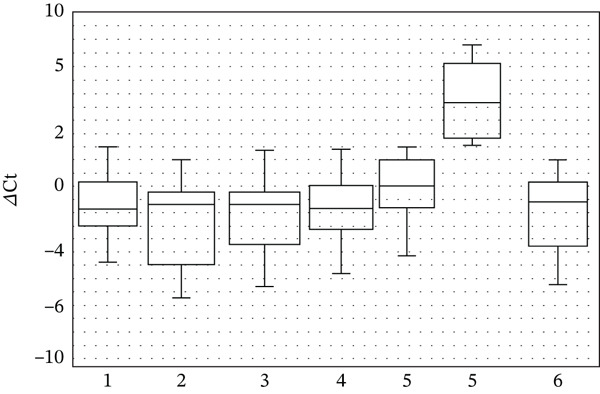
The level of JUN gene transcription in human blood cell cultures relative to GAPDH gene transcription in the presence of plant extracts in the culture medium (1—control; 2—grape seed extract 3.75%; 3—grape seed extract 5.0%; 4—saffron extract 1.25%; 5 and 6—saffron extract 2.5%). Values represent mean ± SD of three biological replicates, each measured in technical triplicate. Statistical significance was determined using the Mann–Whitney *U* test (*p* < 0.05).

Our results also align with literature indicating that saffron and grape seed phytochemicals act through the Nrf2/ARE pathway [45, 46]. Nrf2 is normally sequestered in the cytoplasm by Keap1, but plant‐derived compounds can disrupt this interaction, allowing Nrf2 to translocate to the nucleus and activate the transcription of antioxidant genes [47–49]. The increased expression of SOD1 (Figure 1) and NFE2L2 (Figure 2) observed in our cultures is consistent with this mechanism. Polyphenols in grape seed extract are known to activate MAPK/ERK signaling, leading to phosphorylation of transcription factors and enhanced antioxidant gene transcription [46, 50–52]. The significant upregulation of JUN (Figure 3) in our study supports this pathway, as JUN is a key component of the AP‐1 transcription factor complex. Likewise, saffron carotenoids have been reported to activate Nrf2 via AMPK phosphorylation, further supporting our findings [53]. Together, these pathways explain the observed modulation of antioxidant gene expression in response to the extracts.

Interestingly, donor‐dependent variability was noted in the transcriptional response, particularly for SOD1 and NFE2L2. This heterogeneity may reflect genetic or epigenetic differences in redox regulation among individuals, a phenomenon also described in other studies of phytochemical antioxidants [54]. Despite this variability, the overall trend confirmed that both extracts enhance antioxidant defenses at the transcriptional level.

**Figure 4:**
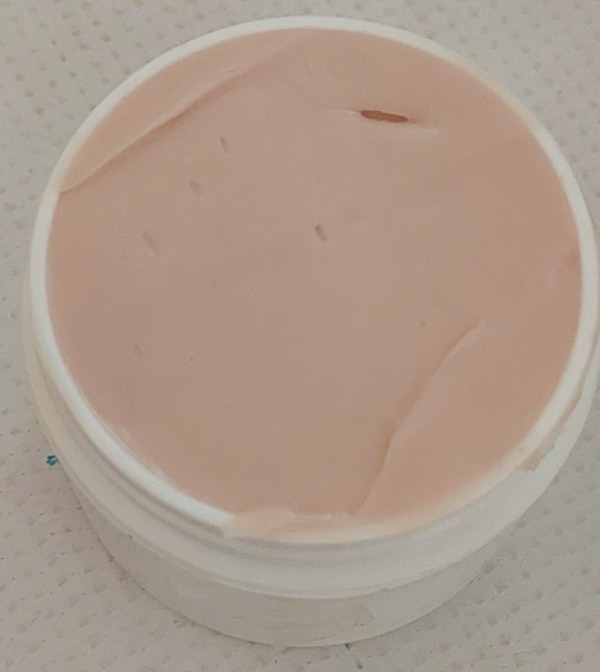
The water/oil antioxidant cream containing saffron extract 2.5% and grape seed extract 5.0%.

From a formulation perspective, the cream containing 2.5% saffron and 5.0% grape seed extract exhibited desirable pharmaceutical properties. It was stable for at least 3 months without phase separation, had a skin‐compatible pH of 5.9, and showed no irritancy in preliminary testing. Its viscosity (31869 centipoise) and spreadability (24.32 g/cm^3^/s) indicate ease of application and consumer acceptability. The visual appearance of the final cream is shown in Figure 4, confirming its homogeneity and suitability for topical use.

The active ingredients in the cream were shown to increase the gene expression of Nrf2, the main regulator of the antioxidant response, and to stimulate the cellular defense program. This is achieved by encoding the enzyme SOD1, which neutralizes superoxide radicals that break down collagen and cause photoaging [55]. Simultaneously, increased JUN gene expression is associated with increased expression of metal matrix metalloproteinases (MMPs), which are directly linked to preventing collagen breakdown [13]. Furthermore, the Nrf2‐SOD1 pathway is essential for maintaining redox balance in dermal fibroblasts, thus reducing the level of skin aging [56]. In the same vein, activation of this pathway is associated with a reduction in inflammatory activity, inhibition of lipid peroxidation in cell membranes, and maintenance of the integrity of the skin barrier [57]. These attributes are essential for translating phytochemical activity into a practical dermal product.

In summary, this study confirms that saffron and grape seed extracts reduce oxidative stress and modulate redox‐sensitive gene expression in vitro, while also demonstrating that these extracts can be successfully incorporated into a stable, skin‐compatible cream. By integrating phytochemistry, molecular biology, and formulation science, our work extends previous findings and provides a foundation for the development of novel antioxidant creams aimed at protecting the skin from oxidative damage and promoting rejuvenation.

## Funding

This study was supported by Ministry of Science and Education FENW, 2026‐0009.

## Conflicts of Interest

The authors declare no conflicts of interest.

## Data Availability

The data that support the findings of this study are available from the corresponding author upon reasonable request.
